# Developing a Plan for the Sustainable Implementation of an Electronic Health Intervention (Partner in Balance) to Support Caregivers of People With Dementia: Case Study

**DOI:** 10.2196/18624

**Published:** 2020-06-25

**Authors:** Hannah Liane Christie, Lizzy Mitzy Maria Boots, Kirsten Peetoom, Huibert Johannes Tange, Frans Rochus Josef Verhey, Marjolein Elizabeth de Vugt

**Affiliations:** 1 Department of Psychiatry and Neuropsychology and Alzheimer Centre Limburg School for Mental Health and Neurosciences Maastricht University Maastricht Netherlands; 2 Department of Family Practice CAPHRI School for Public Health and Primary Care Maastricht University Maastricht Netherlands

**Keywords:** dementia, caregiving, eHealth, implementation, business modeling

## Abstract

**Background:**

Given the increasing use of digital interventions in health care, understanding how best to implement them is crucial. However, evidence on how to implement new academically developed interventions in complex health care environments is lacking. This case study offers an example of how to develop a theory-based implementation plan for Partner in Balance, an electronic health (eHealth) intervention to support the caregivers of people with dementia.

**Objective:**

The specific objectives of this study were to (1) formulate evidence-based implementation strategies, (2) develop a sustainable business model, and (3) integrate these elements into an implementation plan.

**Methods:**

This case study concerns Partner in Balance, a blended care intervention to support the caregivers of people with dementia, which is effective in improving caregiver self-efficacy, quality of life, and experienced control. The large-scale implementation of Partner in Balance took place in local dementia case-management services, local care homes, dementia support groups, and municipalities. Experiences from real-life pilots (n=22) and qualitative interviews with national stakeholders (n=14) were used to establish an implementation plan consisting of implementation strategies and a business model.

**Results:**

The main finding was the need for a business model to facilitate decision-making from potential client organizations, who need reliable pricing information before they can commit to training coaches and implementing the intervention. Additionally, knowledge of the organizational context and a wider health care system are essential to ensure that the intervention meets the needs of its target users. Based on these findings, the research team formulated implementation strategies targeted at the engagement of organizations and staff, dissemination of the intervention, and facilitation of long-term project management in the future.

**Conclusions:**

This study offers a theory-based example of implementing an evidence-based eHealth intervention in dementia health care. The findings help fill the knowledge gap on the eHealth implementation context for evidence-based eHealth interventions after the trial phase, and they can be used to inform individuals working to develop and sustainably implement eHealth.

## Introduction

### Dementia and Caregiving

The combination of an aging population and declining birth rate is proving to be a great challenge for many modern health care systems, resulting in rising costs and spending cuts [1]. In particular, policy makers express concerns about the rising costs of dementia care, as there are currently 50 million people with dementia, and this number is set to triple by 2050 [2]. Informal caregivers of people with dementia, such as spouses, friends, and other loved ones, provide a large part of the necessary care for people with dementia at home [3]. However, the informal caregiving process often results in chronic stress, leading to caregiver overburden, depression, and anxiety [4].

### Electronic Health as a Potential Solution

Policy makers and governing bodies have expressed enthusiasm for electronic health (eHealth) as a solution to tackle these current health care challenges [5,6]. Various eHealth interventions have shown evidence of effectiveness at improving outcomes for the caregivers of people with dementia, such as self-efficacy and dementia knowledge, as well as reduced depressive and anxious symptoms [7-13]. eHealth interventions are defined as “treatments, typically behaviorally based, that are operationalized and transformed for delivery via the internet” [14]. eHealth interventions provide specific advantages to the caregivers of people with dementia, as they can be personalized and adapted to the stage of dementia and allow caregivers to receive psychoeducation without leaving the person with dementia home alone and to seek help without facing the stigma associated with dementia. For these reasons, eHealth is also mentioned as an important part of the Dutch *Deltaplan Dementie* [15] and in the council of the European Union’s dementia policies [16]. Of course, there are also specific challenges associated with implementing eHealth for the caregivers of people with dementia, including the advanced age of many caregivers. While many older adults show high digital literacy, impaired motor, cognitive, and perceptive abilities can constitute relevant barriers [17-19].

### Implementing eHealth for the Caregivers of People With Dementia

Unfortunately, the implementation of evidence-based eHealth interventions into routine practice has proven challenging [20-22], and previous research has shown that very few eHealth interventions for dementia are implemented into practice [23]. Here, implementation is defined as “the process of putting to use or integrating evidence-based interventions within a setting” [24]. A lack of insight into eHealth interventions’ contextual determinants and process changes is an important factor in the slow implementation of many eHealth interventions [25]. Additionally, challenges in implementing eHealth include limited evidence of the demonstrable effects on improving health care outcomes, skeptical attitudes from health care professionals, lack of coordination and management of interventions within health care organizations, and the often peripheral position of potential end users in eHealth development [26]. Many of these issues result from problematic atheoretical implementation and insufficient implementation strategies [27], which are “methods or techniques used to enhance the adoption, implementation, and sustainability of a clinical program or practice” [28]. This lack of successful implementation is an important missed opportunity for the health care system, as advantages of eHealth interventions for health care include the potential to widen access to more remote areas, lower thresholds for participation, improve quality through increased opportunities for personalization, improve service efficiency, and reduce costs [22,29]. To facilitate the sustainable success of these promising interventions, it has been argued that the development of a business model is paramount [30]. Here, a business model is defined as “the rationale of how an organization creates, delivers, and captures value” [31]. Business modeling can be seen as part of an effective implementation strategy, primarily through its potential to both aid sustainable financing and identify value drivers to ensure the relevance of the interventions to the target users [32]. Finally, it is important to note that the specific challenges experienced in implementing eHealth interventions for the caregivers of people with dementia can differ across settings. In this study, we explored the at-home setting of Partner in Balance, which is implemented through health care organizations.

### The Intervention: Partner in Balance

One example of an eHealth intervention to support the caregivers of people with dementia is Partner in Balance. Partner in Balance is a web-based tool to support the caregivers of people with dementia at home, which is applied in a “blended” 8-week eHealth intervention. This “blended” aspect entails that Partner in Balance is delivered through a coach. These coaches are part of participating health care organizations (for example, dementia case management organizations), who have agreed to offer the Partner in Balance intervention to their clients. Partner in Balance coaches are required to have experience (1) in healthcare and (2) with dementia. The coaches are required to take part in a 2-hour Partner in Balance training course, were the intervention is presented and the coaches take part in various coaching exercises.

Afterwards, caregivers first meet coaches face-to-face for an intake session, where relevant modules are chosen to help the caregivers adapt to their new role. At home, the caregivers complete the chosen modules, which consist of caregiver tips, video vignettes, self-reflective assignments, and web-based feedback from the coach. Finally, the coaches and caregivers meet for an in-person evaluation session. Partner in Balance is currently available in Dutch, French, German, and English. The development and testing of Partner in Balance made use of the stepwise approach of the Medical Research Council (MRC) framework for complex interventions [33]. Information on the results of the needs assessment [34], pilot study [35], randomized controlled trial [36], and process evaluation [37] has been published previously. These last two studies showed that Partner in Balance increased caregiver self-efficacy, sense of competency, and quality of life, and was positively evaluated by both caregivers and coaches.

### Aims and Objectives

This paper describes the implementation of Partner in Balance (an evidence-based eHealth intervention) as a use case to inform developers of other evidence-based eHealth interventions for the caregivers of people with dementia. Using insights from real-life pilots and stakeholder interviews, the aim of this study was to shed more light on the implementation context and aid future researchers in the implementation of similar interventions. The specific objectives of this study are to (1) formulate evidence-based implementation strategies, (2) develop a sustainable business model, and (3) integrate these elements into an implementation plan.

## Methods

### Explorative Implementation

#### Real-Life Pilots

To acquire this insight, real-life pilot implementations of Partner in Balance in local care organizations were conducted. Here, the goal was to let the organizations implement Partner in Balance at their own discretion, free from the more rigid protocols of a randomized controlled trial. These pilots ran from September 2016 to September 2019. Organizations participating in the real-life pilots were recruited through two channels. First, Partner in Balance was offered as one of the 15 activities through the euPrevent Senior Friendly Communities (SFC) project [38]. In this project, 32 municipalities in the Netherlands, Germany, and Belgium had the option to implement Partner in Balance for free through local care organizations in their communities. Second, in 2017, Partner in Balance won the Dutch ZonMw *Medical Inspirer Prize* [39], resulting in public attention on the intervention and a small budget to implement Partner in Balance in interested organizations for a limited time. During the real-life pilots, data were collected on the number and type of participating organizations, as well as the number of active coaches and participants.

#### Stakeholder Interviews

From April to June 2019, 14 semistructured qualitative interviews were conducted with stakeholders from patient organizations (n=2), a municipality implementing Partner in Balance (n=1), dementia case management organizations (n=2), mental health care providers (n=3), an eHealth expertise center (n=1), health insurers (n=3), an academic hospital (n=1), and a care research funding body (n=1). These interviews were all conducted in the Netherlands and in Dutch (10 in person and four via Skype). Participants signed an informed consent form. The interviews were recorded and transcribed verbatim. Two researchers (HLC and LMMB) applied inductive thematic analysis by independently coding the transcripts and subsequently grouping these codes into higher level categories and themes [40]. A meeting was held with a third researcher (MEdV) to discuss differences in coding and to reach a consensus. The stakeholder interview questions can be found in Multimedia Appendix 1.

### Sustainable Implementation

#### Overview

**Figure 1:**
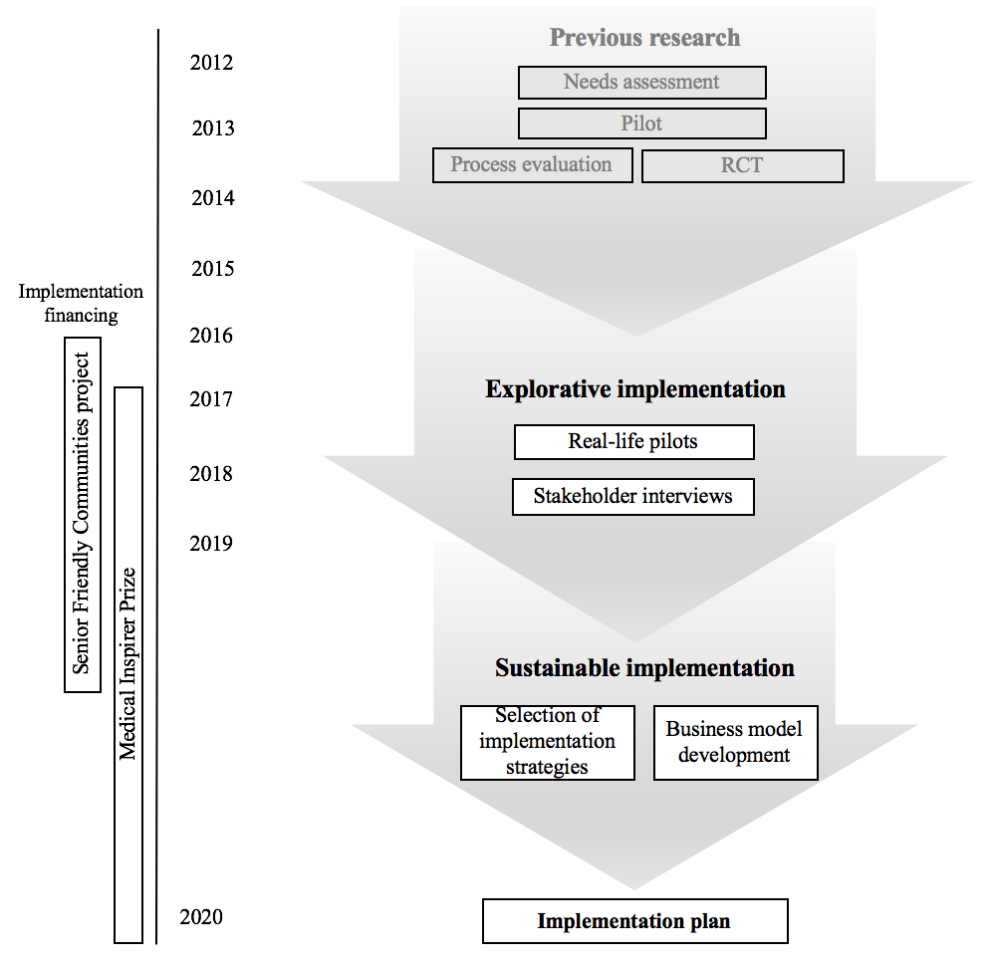
Partner in Balance development and implementation trajectory. RCT: randomized controlled trial.

The goal of this project was to develop an implementation plan based on the information gathered during the explorative implementation. Figure 1 presents an overview of the Partner in Balance implementation trajectory.

#### Selection of Implementation Strategies

The first step in the development of the sustainable implementation plan was the selection of implementation strategies. Based on the information acquired in the previous research and explorative implementation phases, these strategies were formulated by researchers on the Partner in Balance implementation team, which consisted of researchers, the software partner, a coach organization, and the Knowledge Transfer Office.

The selection of strategies was guided by the consolidated framework for implementation research (CFIR) [41]. The CFIR is an established framework for mapping implementation and for eHealth interventions [42]. The CFIR aims to describe determinants, which can serve as barriers and facilitators, that affect implementation outcomes. The CFIR is made up of five domains (intervention characteristics, the outer setting, the inner setting, characteristics of individuals, and the process), containing 39 implementation constructs. The CFIR has been used both to retrospectively evaluate implementation and to prospectively design future implementation strategies [43].

#### Development of a Business Model

The second component of this sustainable implementation plan was the development of a business model. The Partner in Balance business model was developed using the business model canvas [31]. The business model canvas is a popular framework that aims to develop new and document existing business models by mapping the value proposition, key activities, key resources, key partners, cost structure, customer relationships, distribution channels, and revenue of a product or service. The business model canvas has often been used to map business models for eHealth [32,44-46]. The Partner in Balance business model canvas was codeveloped and its face validity was jointly assessed with the involved software partner. The model was iteratively adapted by both parties in reaction to feedback from potential clients. This was done to be able to offer participating organizations some certainties concerning the intervention’s future availability and pricing, as this had already been reported in previous trials as a barrier to adopt the intervention in the future [37].

### Ethical Approval

Ethical approval for these studies was granted by the Medical Ethical Oversight Commission of Maastricht University (approval number 2018-0489).

## Results

### Overview

The following section describes this study’s findings from explorative implementation (real-life pilots and stakeholder interviews), while the subsequent section describes how these findings are integrated to achieve this study’s objectives concerning sustainable implementation (devising implementation strategies and a business model). The final section integrates these findings into a concrete implementation plan.

### Explorative Implementation

#### Real-Life Pilots

Four and a half full-time equivalent researchers worked part-time on the implementation of the Partner in Balance project, recruiting organizations, providing technical and implementation support, managing relationships with organizations and the technology partner, planning and carrying out coach training, and developing new content modules. In the context of the SFC project, three municipalities in the Netherlands, one municipality in Belgium, and one municipality in Germany chose to implement Partner in Balance in their communities. The remaining 27 municipalities (84%) in the SFC project chose to implement other projects. In the context of the Medical Inspirer Prize, 19 organizations chose to implement Partner in Balance for their clients. Table 1 provides an overview of some characteristics of the real-life pilots, including the finding that not all trained coaches ended up coaching participating caregivers.

**Table 1:** Overview of real-life pilots.

Real-life pilot characteristics			Value, n
**Type of organization**			22
	Hospital	6	
	Company	1	
	Municipality	5	
	Case management organization	4	
	Mental health care organization	4	
	Care home	3	
	International research project	1	
Total number of trained coaches			128
Total number of coached participants			122
Average number of participants per organization			10
Average number of coaches per organization			7

#### Stakeholder Interviews

The inductive interview analysis of interviews with potential stakeholders (n=14) resulted in five themes, with their own categories and subcategories (Table 2). The aim of the interviews was to gain insights into stakeholders’ views on barriers and facilitators to the sustainable implementation of Partner in Balance.

**Table 2:** Inductive interview themes.

Theme	Category (*examples of answers*)
1. Future of Partner in Balance	1.1. Good content (*self-management, sustainability, blendedness, personalized, evidence-based, and positive health*) 1.2. Need for Partner in Balance (*not suited for everyone, addition to offline services, psychoeducation, Partner in Balance is needed, an opportunity for a research project to grow, meets caregivers’ needs, and digital factor is a challenge*) 1.3. Extra Partner in Balance functions (*modules for new populations, extra workshops, chat function, more structural support, reminders when inactive, facilitator/contact person, forum, return meetings, and no changes necessary*)
2. eHealth experiences	2.1. Lack of suitable options 2.2. Works better in younger adult populations 2.3. Good investment 2.4. Easier than physical services 2.5. The provider has to be pushing the implementation 2.6. Important to some health insurers 2.7. Not often user-friendly
3. Caregiving context	3.1. Caregiving support (*where can caregivers go for support, often still new for care teams, importance of case management, and financing of caregiving support*) 3.2. Policy
4. Financial context	4.1. Financing models (*public money, subscriptions, and licensing*) 4.2. Potential financers (*caregiver, organization, municipality, labor market, and health insurer*)
5. eHealth implementation process	5.1. Purchase process (*pilots by providers, importance of municipality policy and budget, collaboration with organizations, and decision levels*) 5.2 Evaluation criteria (*financial plan, form of eHealth, who is the eHealth owner, connection to research, and necessary information*) 5.3 Outcomes of success (*waiting lists for care support go down, caregivers satisfied, less case management hours, more referrals, more caregivers supported, more caregivers able to safely live at home, positive real-time evaluations, more care efficiency, and acquisition of cost-effectiveness data*)

### Future of Partner in Balance

This first theme concerns the views stakeholders had on what was good about Partner in Balance and what could be improved in the future. The first category of this theme “good content” showed that all groups of stakeholders had positive attitudes toward the Partner in Balance content and thought many of its components were useful and timely. The second category refers to how stakeholders (especially policy makers and health care professionals) thought that Partner in Balance met caregiver needs, but emphasized that they saw it as complementary to and not as a substitution of face-to-face caregiving services. In the final category of this theme, stakeholders suggested options for additional Partner in Balance functions. These mostly centered around more contact and support, either online (through chat functions and forums) or offline (through meetings, symposia, and a contact person).

### eHealth Experiences

The second theme discusses what stakeholders mentioned concerning the broader eHealth context. In the experience of health care professionals, eHealth is rather difficult to implement, especially in older populations. They also felt that the topic of eHealth was important to health insurers, and the implementation often needed to be pushed by the eHealth provider. Several groups of stakeholders mentioned that eHealth is often not very user-friendly and saw this as an important barrier.

### Caregiving Context

In the third theme “caregiving context,” stakeholders sketched the context in which dementia caregiving support usually takes place, as well as the associated challenges. These challenges included health care professionals’ unfamiliarity with the topic, as well as the importance of case management and how it (and dementia caregiver support in general) is organized. In terms of policy, a trend emerged across the different stakeholders. For policy makers, health care professionals, and health insurers, policy tended to focus less on caregiving and more on self-management, personalization, and positive health. These policy trends were in line with the Partner in Balance content, and this match between the intervention and current policy trends was considered a notable intervention selling point.

### Financial Context

The fourth theme groups stakeholders’ views on the financial context of Partner in Balance. This included responses from policy makers on whether it was ethical to market an intervention developed with public money, as well as different options and calculations for various subscription and licensing models. Regarding the latter, large variations in the suggested price were observed, with caregiver contributions of €0 (US $0), €1 (US $1), €25 (US $28), or €35 (US $40) for a full course (as a way to ensure adherence) and €200 (US $226) to €700 (US $791) paid by care organizations (including the costs of training, coaching hours, and hosting). However, the majority of stakeholders did not think that informal caregivers should be the ones paying for the intervention, but rather that this should fall to the care organizations, municipalities, or health insurers (no stakeholders suggested the intervention be somehow free for all parties). The health care professionals favored a yearly subscription model, where organizations could buy licenses for the desired number of participants. In the second category “potential financers,” the Dutch national health care insurance system and how it relates to the municipal prevention mandate were the main topics of discussion. In particular, the classification of Partner in Balance as a tool for prevention (as buying a license could then be more suited to a municipality) or treatment (as buying a license could then be more suited to a health care insurer) was important. Other potential financing options were interested parties from the labor market (to combat loss of workforce to caregiver burden) and buy-in care networks (where local dementia care organizations group together in care networks).

### eHealth Implementation Process

The final theme groups stakeholders’ statements on the process for their organizations to potentially adopt, disseminate, and implement new eHealth interventions for the caregivers of people with dementia. Concerning the first category “purchase process,” the policy makers emphasized the need for the intervention to be approved at many levels, including in the budget and policy (especially for municipalities), as well as the added value of testing interventions through pilots with local collaborations. A number of evaluation criteria used by the organizations to decide whether to implement an intervention were discussed (Table 2). Most importantly, health insurers repeatedly mentioned the need for data on effectiveness and cost-efficiency. Interestingly, they emphasized that the data could be speculative and qualitative (and not necessarily longitudinal or randomized controlled). Useful outcomes with which health care organizations (such as dementia case managers) could measure implementation success were waiting list reduction, less case management hours, more referrals, more supported caregivers, more caregivers able to safely live at home, positive real-time evaluations, and more care efficiency.

### Sustainable Implementation

#### Selection of Implementation Strategies

The devised implementation strategies were principally aimed at helping integrate Partner in Balance more into the coach organizations, as well as motivating and engaging these coaches and their management more effectively (domains of “inner setting” and “characteristics of the individuals”). This was based on the finding from usage data that not all trained coaches ended up coaching. In order to enhance the attractiveness of Partner in Balance to potential clients, more financial insights into the pricing and long-term business modeling of Partner in Balance were necessary (domain “characteristics of the intervention”). Additionally, strategies were formulated to streamline Partner in Balance administration and project management (domain “process”), as well as to expand and disseminate its use (domain “outer setting”). Table 3 lists the CFIR domains and corresponding implementation strategies.

**Table 3:** Partner in Balance implementation strategies and consolidated framework for implementation research domains.

CFIR^a^ domain	Partner in Balance implementation strategy	Targeted CFIR subdomains	Operationalization
Characteristics of the intervention	Assess Partner in Balance’s effect on an organization’s care costs	Evidence strength and quality, relative advantage, adaptability, and complexity	Determine the intended effect on various aspects as follows: reduced experienced workload, shorter waiting time for case management, lower time investment for case manager, longer estimated period as full-time informal caregiver, less/later requirement for home care, and less/later crisis relief. Comparison of this longitudinal use of health care data during Partner in Balance deployment with a control group for the introduction of Partner in Balance using registered health insurers. Additionally, comparison of baseline measurement and follow-up measurement of maintenance time in a cohort of clients who receive Partner in Balance.
Characteristics of the intervention	Develop more detailed financial models	Cost, trialability, design quality and packaging, and interventions source	Determine the costs of required resources. Compare the necessary case management hours and waiting list before and after implementing Partner in Balance. Map responsible budgets.
Outer setting	Explore integrating Partner in Balance in case management in the Netherlands, as well as outside of Limburg	Cosmopolitanism, patient needs, and resources	Overview of bottlenecks and facilitators to offer Partner in Balance in the Netherlands, as well as outside of Limburg.
Outer setting	Subsidy applications and participation in networking and knowledge sharing events	External policies and incentives, cosmopolitanism, and peer pressure	Subsidy application involving crucial implementation partners in innovation clusters. Overview experiences and lessons learned by other innovation clusters.
Inner setting	Integrate Partner in Balance within Help with Dementia Limburg (case management organization)	Structural characteristics, organizational incentives and rewards, goals and feedback, and readiness for implementation	Prepared supervision plan for new clients in which the Partner in Balance offer is included as a fixed part.
Inner setting	Further development and embedding of inspiration sessions through integration in coach training	Tension for change, relative priority, and access to knowledge and information	Web-based inspiration session including video material in which case managers and caregivers explain the use and added value.
Characteristics of the individuals	Development of content for inspiration sessions and web-based coach training	Knowledge and beliefs about the intervention, self-efficacy, and individual stage of change	Inspiration session content and guide. Web-based coach training content.
Characteristics of the individuals	Pilot inspiration sessions and web-based coach training	Individual identification with the organization and other personal attributes	Custom inspiration session and web-based coach training based on feedback from current coaches.
Process	Evaluate coach training (by participants) plus evaluate web-based training	Reflecting and evaluating, as well as engaging (champions)	Overview of the number of chosen “live” or “online” trainings, including qualitative evaluation by participants on the quality, method, and content of the training.
Process	Disseminate progress	Engaging (formally appointed implementation leaders)	Short progress reports distributed among case managers (Help with Dementia newsletter) and nationwide (Alzheimer NL/dementie.nl).
Process	Write scientific publications and policy reports	Engaging (opinion leaders)	Scientific publications in peer-reviewed professional journals and policy reports (communication to contacts within the Ministry of Health, Welfare and Sport).
Process	Report to the public	Engaging (external agents of change)	Lay report
Process	Organize symposium	Engaging (external agents of change), reflecting, and evaluating	Symposium including communication and feedback of results to the society.
Process	Project coordination	Planning	Overview of project members in lead and coordination tasks.
Process	Define go and no go moments and possible next steps	Executing	Qualitative inventory of existing barriers and facilitators for scaling up and use.

^a^CFIR: consolidated framework for implementation research.

#### Development of a Business Model

**Figure 2:**
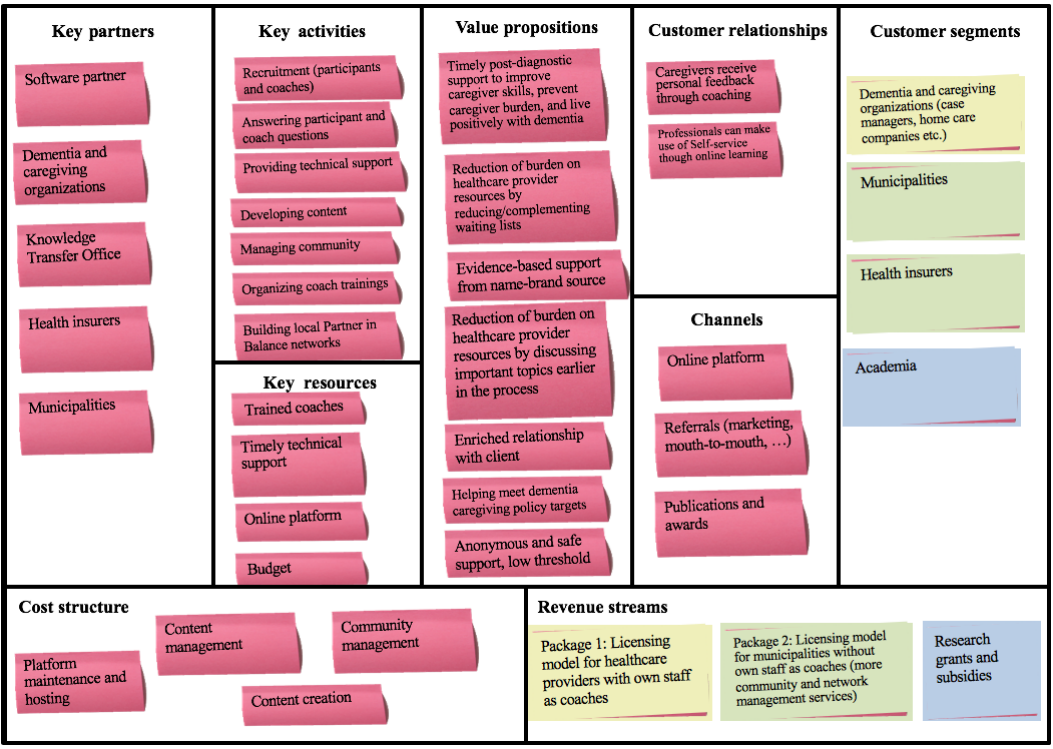
Partner in Balance business model canvas. The nine elements help describe a firm's or product’s structure by mapping its value proposition (middle element), infrastructure, (top left three elements), customers (top right three elements), and finances (bottom two elements).

Figure 2 presents a depiction of how sustainable implementation could hypothetically be achieved based on insights from the previous implementation phases and the stakeholder interviews. Partner in Balance has added value for caregivers, health care organizations, and municipalities (“value propositions”), and together with the “channels” and “customer relationships,” this helped the team form a better view of the intervention’s desirability to potential customers. In the proposed business model, three distinct types of customers were identified (“customer segments”). As a result, it was decided that two of these customer segments required specific licensing models (“revenue streams”). First, health care support providers, such as case management organizations, require no help with recruitment as they can supply their own coaches in house and are targeted with package 1. Second, municipalities are targeted with a package that additionally includes identifying which local organizations can provide coaches (package 2). These revenue streams would in turn finance the main cost drivers of Partner in Balance described in “cost structure,” which are made possible by the “key partners,” “key activities,” and “key resources.” The development of this business model and collaboration with the Knowledge Transfer Office and the software partner are crucial to the sustainability of the implementation plan through its provisions for long-term financing of the Partner in Balance intervention.

### Integration

The six components of the implementation plan are presented in this section. The operationalizations of the targeted CFIR subdomains were combined into components 1 to 5, while the business model canvas led to component 6. Based on these inputs, the components of the integrated plan for the sustainable implementation of Partner in Balance were as follows: (1) a ready-to-use Partner in Balance inspiration and intervision session format (live and online version and online coach training) to stimulate inner setting enthusiasm, (2) a guide for the implementing organization, in which Partner in Balance is included as a fixed offer in the first phase after diagnosis, (3) the writing of a report on financing options and cost-effectiveness, (4) efficient communication of project results through different channels, (5) a coordination plan and division of responsibility, including risk management, and (6) a sustainability plan including a licensing model. This licensing model is currently structured for an organization to pay for coaching one client per payment. The coach’s organization and municipality chooses how many coach licenses they wish to buy. Additionally, implementation costs are charged depending on the type of package desired by the implementing organization.

## Discussion

### Addressing the Lack of Information on Long-Term Financing of the Intervention

In previous research, the Partner in Balance process evaluation [37] reported that initial implementation challenges were related to a lack of financing and time necessary to implement the intervention. The findings from this study’s real-life pilots and stakeholder interviews made it possible to more precisely describe the previously identified issues and devise solutions by constructing a preliminary business model. In this study, stakeholders reported an unwillingness to commit resources to an intervention that might not be available in the future or that they might not be able to afford. This is in line with previous research that also advocated the application of business models to evidence-based interventions to facilitate long-term implementation [32,47]. Thus, this study contributes to literature on the implementation context by providing insights into this important implementation barrier, namely the lack of reliable pricing information for implementing evidence-based eHealth interventions to support the caregivers of people with dementia [48]. Additionally, the implementation strategies developed in this study using the CFIR helped ensure that the different components of successful implementation were considered in the business model.

### Addressing the Lack of Information on the Organizational Context

There has been little research on the perspectives of the parties involved in the real-life implementation of evidence-based eHealth interventions for the caregivers of people with dementia [23]. For this reason, it was necessary to formulate a targeted implementation plan for Partner in Balance, which would help tailor Partner in Balance to this relatively underexplored dementia health care context. The implementation strategies and proposed business model resulted in an implementation plan that aimed to facilitate the integration of this evidence-based intervention into the organizational structures found in clinical practice. In this study, stakeholders in the domains of eHealth and dementia care perceived eHealth as difficult to implement and the usage data showed that 84% of SFC municipalities chose to implement other dementia-related projects instead of Partner in Balance, underscoring the role of organizations as gatekeepers in the implementation of evidence-based eHealth interventions for caregivers of people with dementia. This is in line with previous eHealth research that has cited the unfamiliarity of both implementing staff and the target population with web-based support tools as an important barrier to implementation [17,19]. Additionally, the fact that eHealth circumvents traditional health care delivery structures contributes to the difficulty many care organizations and governing bodies experience in implementing the interventions and adapting existing structures and norms to integrate them [49]. However, in the context of eHealth for dementia, the stakeholders did see Partner in Balance as needed and timely, particularly as it fits into current trending policy targets of self-management, personalization, and positive health, which have been advocated by the literature [50-52]. These findings confirm that it is important to continue to investigate and accommodate the evolving role of dementia care professionals in the context of emerging eHealth innovations and consider embedding eHealth care education into training programs for health care professionals [53,54], as proposed by the strategies integrated into this study’s implementation plan for Partner in Balance.

### Recommendations From the Partner in Balance Case Study to Aid the Implementation of Future eHealth Interventions Supporting the Caregivers of People With Dementia

It is the authors’ aim that the findings presented in this study also inform future eHealth interventions for the caregivers of people with dementia and facilitate more efficient development and implementation. We present the following recommendations based on the lessons learned throughout the various phases of the Partner in Balance implementation:

(1) Health care organizations are often willing to pay for eHealth for their caregivers of people with dementia as long as the price of implementation is set, the evidence base is reliable, and the benefits to the organization are clear.

(2) It is recommended to form an “innovation cluster” with dementia health care institutions (the implementers, such as dementia case management organizations) together with parties who can buy licenses (such as municipalities), while other organizations (such as health insurers) reimburse the health care organization’s staff hours.

(3) eHealth interventions to support the caregivers of people with dementia cannot be implemented as a ready-to-go one-size-fits-all project. Offline guidance and tailoring will always be necessary. Therefore, it is important to budget for this and identify which partners will be a part of the so-called “innovation cluster” to ensure a realistic implementation plan.

(4) Finding a balance between these differing prioritizations and identifying which of the involved parties should be the financer and which should be the implementer in the dementia health care context are challenges best addressed early in the development process (preferably even before the effectiveness trial).

(5) It is important to emphasize to potential eHealth buyers that eHealth should always be complementary to other offline dementia caregiving services and not a replacement of existing face-to-face services.

(6) It is recommended to construct a preliminary business model canvas at the start of implementation (before the effectiveness trial) in order to identify all relevant partnerships, customer relationships, and revenue streams in the local dementia health care context. Doing this will allow researchers to create a product that is attuned to its specific market. If possible, it is also suggested to work with a commercial partner from the start. Using the business model canvas to inform our pricing and implementation plan was very helpful.

(7) Future developers should incorporate an explorative implementation phase after the trial context. It is necessary to flexibly explore different pricing models and iteratively address real-world implementation challenges prior to actually charging organizations.

(8) Using the CFIR helped to formulate implementation strategies targeted at many different aspects of implementation. It was particularly helpful in structuring thinking on project management, as well as the engagement of the implementing organization’s staff and management.

This study has helped fill the knowledge gap concerning the implementation context for eHealth interventions for the caregivers of people with dementia in two important ways. It has added to the existing literature by providing an example of a business model to aid the implementation of an evidence-based eHealth intervention for the caregivers of people with dementia, as well as specific implementation strategies to facilitate integration into the dementia health care context. Future research should evaluate which types of implementation strategies are most successful at achieving long-term implementation. In particular, as concluded from the stakeholder interviews, a more in-depth cost-effectiveness study is needed to encourage more active participation from health insurers and health care organizations.

### Strengths and Weaknesses

This study has unique and important strengths. First, this study made use of well-established theoretical frameworks to guide implementation, using the MRC framework for the development and evaluation of intervention effectiveness, as well as the CFIR and business model canvas. Second, despite its theory-driven approach, this study illustrated a practical and real-word representation of the implementation of an evidence-based eHealth intervention. By iteratively adapting the intervention and being able to adapt with more agility to implementation issues than is normally possible in a strict trial context, this study provided a realistic view of the implementation process and context.

This study also had several weaknesses. First, though it was intended as a “real-world” illustration of bringing an evidence-based eHealth intervention to the market, the actual implementation was still very much dependent on the researchers guiding and facilitating this implementation through the research project. However, this study still provides a useful overview of the steps necessary to construct a realistic implementation plan. Second, several of the interviewed stakeholder had been involved in the Partner in Balance development in the past (four out of 14 stakeholders). This could have resulted in some positive bias to look favorably at the intervention’s future implementation. However, the authors believe it was necessary to include some interview participants who had real knowledge of the working of Partner in Balance. Finally, people with dementia were not included as stakeholders in this study. This is because the intervention was developed together with the caregivers of people with dementia, and it exists in its current form as a result of their needs and wishes. The focus of this study’s stakeholder interviews was on the surrounding implementation context and organizational determinants. Furthermore, the use experience of the intervention from the perspective of caregivers was explored in depth in the Partner in Balance process evaluation [37].

### Future Research Areas

Future research will include an evaluation of the proposed implementation plan. In particular, as concluded from the stakeholder interviews, a more in-depth cost-effectiveness study is needed to encourage more active participation from health insurers and health care organizations.

### Conclusions

Stakeholders saw eHealth as difficult to implement, but as an approach that is needed and timely, particularly as it fits into the current trends of self-management, personalization, and positive health. Applying the CFIR to devise theory-driven implementation strategies was primarily useful for targeting overlooked implementation aspects, such as ensuring effective and sustained engagement of coaches, streamlining project management, expanding and disseminating the intervention, and enhancing insights into pricing and long-term business modeling, in order to ensure sustainability. Insights from business modeling resulted in two different kinds of licensing agreements (one for municipalities and one for organizations). Finally, the authors recommend thoroughly exploring the organizational and health care contexts of the intervention and then forming “innovation clusters” (consisting, for example, of a technology developer, research team, intervention provider, and health insurer or other funder) from the start of eHealth development. This will help ensure that the intervention meets the needs of its target users (both the end users and the implementing staff).

## Appendix

Multimedia Appendix 1 Stakeholder interview questions.

## Abbreviations

CFIR: consolidated framework for implementation research
eHealth: electronic health
MRC: Medical Research Council
SFC: Senior Friendly Communities
