# Supplementary material for: Developing a Plan for the Sustainable Implementation of an Electronic Health Intervention (Partner in Balance) to Support Caregivers of People With Dementia: Case Study
Source: JMIR Aging. 2020 Jun 25;3(1):e18624. doi: 10.2196/18624 (PMC7380981; doi:10.2196/18624)
Supplement: Multimedia Appendix 1 [file aging_v3i1e18624_app1.docx]

**Appendix 1**

**Stakeholder interview questions.**

I. General

Theme 1: Current online informal care support of the organization

1. What is used within this organization about:

a. Informal care support?

b. eHealth in general?

c. What is your experience with this?

2. Process:

a. Who decides within your organization on the purchase of these products?

b. Is there an evaluation process to purchase products? What are the criteria in this process? What information is needed for this decision and payment?

c. How are they paid (with which funds)?

d. What are the barriers to these products

i. to implement

ii. to fund

iii. to use?

Theme 2: Application to Partner in Balance

3. Is there a need for PiB?

4. Who would decide on the purchase of PiB?

5. How would this be funded?

6. What information is needed for this decision?

7. What are the barriers to paying and using PiB?

8. When was the implementation successful? Which outcomes must be met?

Theme 3: Financial

9. How much do you now pay for informal care support / eHealth?

10. Would you pay more for more modules?

11. Would you pay more for different support options?

12. What support options would be needed?

II. Specific for different stakeholders

1. Insurers:

a. What is the experience so far with informal care support within your organization? Which initiatives already exist?

b. How can informal care support be funded / reimbursed (within the premium)?

c. Can online informal care support be funded from the health insurance policy and under what conditions?

d. How much can organizations pay for PiB? What is a better model: organization or participant? Why?

e. Prevention vs. treatment: preference?

2. Municipalities:

a. Can PiB be financed by the municipality / WMO?

b. Who could still finance PiB in this municipality? For example, companies (for their staff)?

c. What would be a better model for you, organization or participant? Why?

3. Clinicians (psychologists and general practitioners) / hospitals:

a. Can the care time (face-to-face) be declared?

b. Can the product itself be financed (independently of the face-to-face care time)

c. Can hospitals link PiB to diagnostics?

d. Management: What could be a realistic price? Can you compare this with existing products for caregivers? Which model (organization, participant) would be better? Why?

e. Clinicians: What could be a realistic price? Can you compare this with existing products for caregivers? What is your experience with this?

4. Funding bodies: What can organizations pay? What is a good / realistic price? Can we compare PiB with other products? If so, what is the price structure of these products?
